# 麦门冬合千金苇茎汤抑制A549细胞增殖作用及其机制

**DOI:** 10.3779/j.issn.1009-3419.2010.05.18

**Published:** 2010-05-20

**Authors:** 宇辉 周, 瑧 詹, 于平 唐, 金廒 段, 旭 张

**Affiliations:** 1 21004 南京，南京中医药大学基础医学院中西医结合基础学科 College of Basic Medicine, Nanjing University of Chinese Medicine, Nanjing 210046, China; 2 210046 南京，江苏省方剂研究重点实验室 Jiangsu Key Laboratory for TCM Formulae Research (LTCMF), Nanjing 210046, China

**Keywords:** 肺肿瘤, 增殖, 凋亡, 信号转导, Lung neoplasms, Proliferation, Apoptosis, Signal transduction

## Abstract

**背景与目的:**

采用抗肿瘤中药治疗恶性肿瘤是具有中国特色的肿瘤治疗方式。本研究旨在体外筛选出麦门冬合千金苇茎汤抑制人肺癌A549细胞增殖的有效部位，并对其进行分子机制探讨。

**方法:**

通过MTT法和细胞克隆形成试验体外初步筛选出麦门冬合千金苇茎汤抑制A549细胞增殖的有效部位；流式细胞仪进行细胞周期分析；Hoechst 33258染色观察凋亡形态的变化；Western blot对肺癌相关通路进行检测。

**结果:**

乙酸乙酯萃取部位可显著抑制肺癌A549细胞的生长，且对正常细胞HFL-1无明显影响；与对照组相比，10 μg/mL乙酸乙酯萃取部位作用后克隆形成抑制率达73.86%（*P* < 0.01），流式检测凋亡率为33.86%（*P* < 0.01），荧光显微镜下可见凋亡形态学特征；抗凋亡蛋白EGFR和ERK表达显著下调（*P* < 0.01）。

**结论:**

麦门冬合千金苇茎汤乙酸乙酯萃取部位能显著抑制A549细胞的生长，并通过下调EGFR/ERK信号转导通路诱导细胞凋亡，因此应进一步分离，明确复方抗癌作用的物质基础。

## Abstract (en)

**Background and objective:**

Traditional Chinese medicine is an approach for malignant tumor treatment with Chinese characteristics. The aim of this study is to investigate the inhibitory effects of Maimendong & qianjinweijing decoction extract on A549 human lung cancer cell line proliferation and explored its probable molecular mechanisms.

**Methods:**

A549 cells were treated with drugs in different does and time. The effects on the proliferation of A549 cells were detected by MT assay and clonogenic assay *in vitro*. Cell cycle was analyzed by?ow cytometry. Morphological changes of the apoptosis of cancer cells were observed by Hochest 33258 staining. Western blot was performed to detect apoptosis-related gene expression.

**Results:**

Ethyl acetate extract inhibited the growth of A549 cells but not in HFL-1 cells. Compared with controls, administration of 10 μg/mL ethyl acetate extract resulted in 73.86% decrease in colony formation (*P* < 0.01), apoptotic rates of 33.86% (*P* < 0.01), and morphological changes of apoptosis in A549 cells. The expression of anti-apoptotic protein EGFR and ERK were signifcantly down-regulated (*P* < 0.01).

**Conclusion:**

Ethyl acetate extract might inhibit proliferation and induce apoptosis in A549 cells via downregulation of EGFR/ERK signal transduction pathway. Terefore, ethyl acetate extract should be further separated in order to identify the material fundamentals on anti-cancer effect.

肺癌是当前发病率和死亡率增长最快的恶性肿瘤，对放疗、化疗和免疫治疗的敏感性均较差，寻找有效的治疗方法是当前肺癌治疗亟需解决的问题。采用抗肿瘤中药治疗恶性肿瘤是具有中国特色的肿瘤治疗方式。麦门冬合千金苇茎汤分别是《金匮要略》记载的主治肺痿肺痈的中医古方，麦门冬汤主治“肺中气阴亏虚”、千金苇茎汤清肺化痰活血，两方合用，具有清热养阴、润肺降气、祛瘀排脓之功，与现代中医所认为之肺癌的主要病机“气阴两虚、热毒痰瘀互结”^[1]^基本相符。现代也有人以其中一首方剂加味的方式应用于临床来治疗肺癌，本研究以人肺腺癌细胞系（A549）为主要对象，研究麦门冬汤合千金苇茎汤有效部位群对肺癌细胞株的抑制作用及可能的分子机理。

## 1 材料与方法

### 1.1 材料

#### 1.1.1 细胞系

肺癌细胞株A549和人胚肺成纤维细胞HFL-1均购自中科院上海生物细胞所。

#### 1.1.2 药物及处理

麦门冬汤合千金苇茎汤萃取部位，由南京中医药大学方剂重点实验室制备，将麦门冬合千金苇茎汤（原药材总计21.6 kg）进行水煎煮并挥发油提取，得到挥发油部分和水提液部分。将水提液浓缩后醇沉，醇溶液回收溶剂至无醇后，依次用环己烷、乙酸乙酯、正丁醇萃取，最后共得到8个部分样品，样品均配制成100 mg/mL的母液，-20 ℃保存，临用前解冻。

#### 1.1.3 主要试剂

培养液RPMI-1640为美国GIBCO公司产品；优级新生牛血清为杭州四季青生物制品厂产品；胰蛋白酶为AMRESCO公司产品；二甲基亚砜（dimethylsulfoxide, DMSO）为AMRESCO公司产品；噻唑蓝[3-(4, 5-dimethylthiazol-2-yl)-2, 5-diphenyl tetrazoliumbromide, MTT]为AMRESCO公司产品；吉姆萨染液购自南京建成生物工程研究所；碘化丙啶（propidium iodide, PI）和RNaseA购自美国Sigma公司；Hoechst 33258染色剂为碧云天生物技术研究所产品；抗EGFR抗体和抗ERK1/2抗体购自美国Santa Cruz公司；抗β-actin抗体购自美国GenScript公司；辣根过氧化物酶偶联羊抗兔和羊抗鼠IgG抗体均购自美国Immunology Consultants Laboratory公司；化学发光试剂ECL购自碧云天生物技术研究所；其余试剂均为国产分析纯。

#### 1.1.4 主要仪器及设备

CO_2_细胞培养箱（美国Thermo Scientific Forma），倒置显微镜（美国Olympus），酶标仪（美国Bio-Rad），FACScan型流式细胞仪（美国Becton Dickinson），Z-32K型高速冷冻离心机（德国HERMLE），-70 ℃ 309L型低温冰箱（日本SANYO），电泳仪（美国Bio-Rad）。

### 1.2 方法

#### 1.2.1 细胞培养

人非小细胞肺癌细胞A549和人胚肺细胞HFL-1用含10%小牛血清的RPMI-1640（青霉素100 U/mL、链霉素100 μg/mL，pH7.4）培养液，置于37 ℃、5%CO_2_培养箱中培养，取对数生长期的细胞进行实验，分别用不同浓度的部位样品处理细胞，未加药组为对照组。

#### 1.2.2 细胞增殖活性测定

采用噻唑蓝（MTT）比色法。取对数生长期的A549细胞和HFL-1细胞以每孔5×10^3^个细胞的密度接种于96孔板，设对照组和不同部位浓度处理组（1 μg/mL, 10 μg/mL, 100 μg/mL），每组6个复孔。于作用后72 h，每孔加入MTT（5 mg/mL）20 μL，37 ℃下继续培养4 h，弃去上清，加入DMSO溶解蓝紫色颗粒后，以Ascent酶标仪检测波长490 nm测定吸光度值（*A*值），实验重复3次，按以下公式计算细胞生长抑制率。细胞存活率（%）=实验组平均*A*值/对照组平均A值×100%。

#### 1.2.3 形态学观察

取对数生长期的A549细胞以每孔5×10^3^个细胞接种于96孔板，培养过夜，于倒置显微镜下观察不同部位分别作用A549细胞后24 h、48 h及72 h时细胞形态变化。

#### 1.2.4 集落形成测定

采用平板克隆形成实验。取对数生长期的A549细胞，以250个细胞/孔均匀接种于6孔板中，培养过夜，使细胞贴附，分别加入用含10%小牛血清的RPMI-1640培养液稀释的终浓度为1 μg/mL、10 μg/mL、100 μg/mL的部位样品，每组3个平行孔，置37 ℃、5%CO_2_培养箱中培养7天，去除培养液，PBS洗2次，95%乙醇固定10 min，吉姆萨染液染色10 min，自来水冲洗，晾干，用Quantity One软件计算克隆数，按以下公式计算克隆形成率和克隆形成抑制率。克隆形成率（%）=克隆数/接种细胞数×100%；克隆形成抑制率（%）=1-（实验组克隆数/对照组克隆数）×100%。

#### 1.2.5 细胞生长周期分析

采用PI染色检测细胞周期。收集药物作用72 h的A549细胞，用PBS洗涤，1 000 rpm、10 min离心2次，将细胞沉淀充分混匀，缓慢加入1 mL冰乙醇（70%）固定保存于-20 ℃。检测前PBS洗涤去除乙醇，1 200 rpm、10 min离心2次，加入PI（50 μg/mL）染液混均，4 ℃避光孵育30 min后，用流式细胞仪（美国Becton Dickinson公司）进行DNA含量和细胞周期分析。所用软件为Cellqest，低于G_1_期的细胞（亚G_1_期）为凋亡细胞，其占细胞总数的比例为凋亡细胞比例。

#### 1.2.6 凋亡的细胞核形态学检测

采用Hoechst 33258荧光染色。取对数生长期的A549细胞，均匀接种于预置盖玻片的6孔板中，培养24 h后，分别以RPMI-1640培养液稀释的终浓度为1 μg/mL、10 μg/mL、100 μg/mL的部位处理，同时设对照组，72 h后，吸弃培养液，进行Hochest 33258染色，方法参照说明书进行，染色后荧光显微镜下观察细胞凋亡的形态学改变。结果判断：正常细胞的细胞核呈现弥散均匀荧光，出现细胞凋亡时，细胞核或细胞质内可见浓染致密的颗粒块状荧光，甚至可见DNA荧光碎片。

#### 1.2.7 Western blot检测

用裂解液处理收集细胞，以蛋白提取样品作SDS-PAGE，电泳后转膜，封闭后加入一抗（抗体稀释浓度为1:200），PBST洗膜3次，每次10 min；加入二抗工作液（稀释浓度为1:5 000），PBST洗膜3次，每次10 min；浸于适量ECL化学发光试剂中（A液:B液=1:1）1 min，取出，室温条件下用保鲜膜包置于暗盒中，压片曝光30 s-3 min，洗片，用Quantity One软件进行灰度分析。

### 1.3 统计学分析

采用统计软件SPSS 15.0行统计分析，所有定量数据均以Mean±SD表示，组间比较采用单因素方差分析，以*P* < 0.05为差异有统计学意义。

## 2 结果

### 2.1 MTT比色法检测的结果

麦门冬汤合千金苇茎汤8个提取部位分别作用A549细胞72 h后，第6部位即乙酸乙酯萃取部位各浓度细胞存活率明显降低，有统计学差异（*P* < 0.01），提示乙酸乙酯萃取部位有明显抑制细胞生长的作用，10 μg/mL和1 μg/mL乙酸乙酯萃取部位作用的细胞存活率 < 50%，引起的细胞生长抑制作用明显大于100 μg/mL乙酸乙酯萃取部位（*P* < 0.05）（图 1）。

**1:**
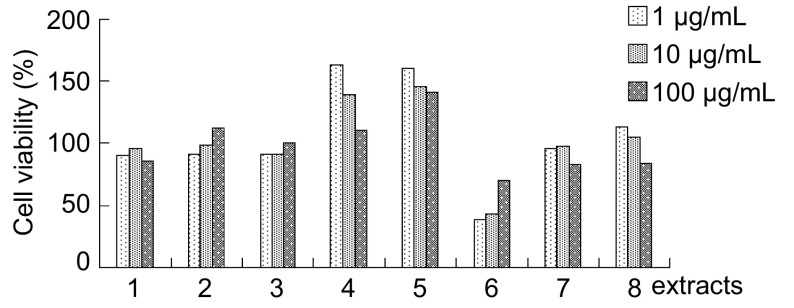
麦门冬汤合千金苇茎汤8个部位对A549细胞生长的影响 The effect of eight extracts form Maimendong & qianjinweijing tang on A549 cells growth

### 2.2 不同浓度乙酸乙酯萃取部位对A549细胞和HFL-1细胞生长的影响

作用72 h后，不同浓度的乙酸乙酯萃取部位均有促进HFL-1细胞生长和抑制A549细胞的作用，同一浓度，HFL-1细胞存活率明显高于A549细胞存活率，以10 μg/mL乙酸乙酯萃取部位组最为明显，引起A549细胞生长抑制作用明显高于100 μg/mL（*P* < 0.01）；同时，促进HFL-1细胞生长作用明显高于其他浓度的药物（*P* < 0.01）（图 2）。

**2:**
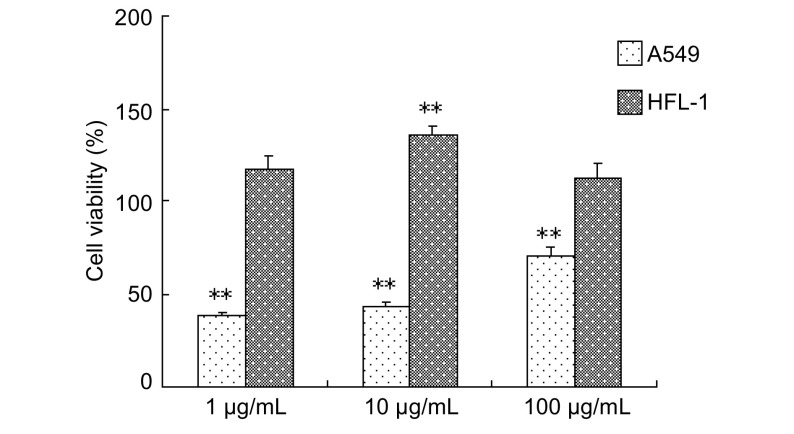
乙酸乙酯萃取部位对细胞生长的影响 The effect of ethyl acetate extract on cells growth

### 2.3 细胞形态学观察结果

在倒置显微镜下可见正常对照组细胞生长旺盛，分布密集，多呈多角形，细胞走向趋于一致，多角形细胞的细胞体饱满、有2-3个扁平而长的突起，细胞核较大，呈卵圆形且多居中。用乙酸乙酯萃取部位处理A549细胞后，随着作用时间的延长，细胞间隔增宽，突起缩短。细胞质减少，细胞体缩小，黑色颗粒增多（图 3）。

**3:**
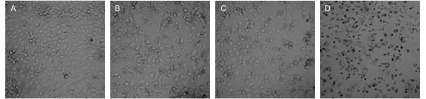
不同浓度乙酸乙酯萃取部位对A549细胞增殖的影响（×200） Proliferative inhibition of different concentrations of ethyl acetate extract on A549 cells (×200). A: Control group; B: 1 μg/mL ethyl acetate extract group; C: 10 μg/mL ethyl acetate extract group; D: 100 μg/mL ethyl acetate extract group.

### 2.4 对A549细胞集落形成的影响

经不同浓度乙酸乙酯萃取部位处理后的细胞克隆形成数明显减少（图 4），说明乙酸乙酯萃取部位抑制了A549细胞的克隆形成。10 μg/mL乙酸乙酯萃取部位组较对照组克隆形成率明显降低，克隆形成抑制率显著升高，克隆形成抑制率可达 > 70%（*P* < 0.01）（表 1）。

**4:**
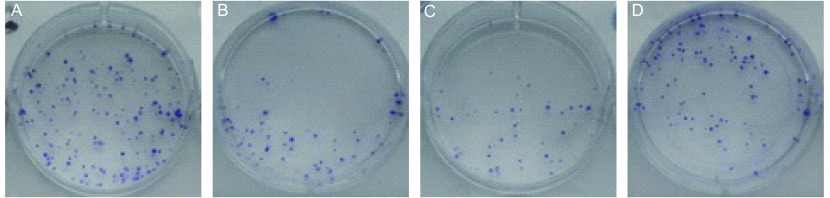
不同浓度乙酸乙酯萃取部位处理A549后细胞克隆形成情况 The effect of different concentrations of ethyl acetate extract on colony formation of A549 cells. A: Control group; B: 1 µg/mL ethyl acetate extract group; C: 10 µg/mL ethyl acetate extract group; D: 100 µg/mL ethyl acetate extract group.

**1:** 不同浓度乙酸乙酯萃取部位处理A549细胞后克隆形成率及克隆形成抑制率的比较 Comparion of clone-formation rate and clone-formation inhibition rate among A549 cells treated with different concentration of ethyl acetate extract

Ethyl acetate extract	1 µg/mL	10 µg/mL	100 µg/mL
Rate of clone-formation (%)	22.40	25.20	64.80
Inhibition rate of clone-formation (%)	76.76	73.86	32.78

### 2.5 细胞周期分析

流式细胞术检测结果见表 2，10 μg/mL乙酸乙酯萃取部位作用后细胞周期与对照组的细胞相比细胞周期的分布无明显改变（*P* > 0.05）。与对照组（图 5A）比较，实验组G_1_期峰前出现显著的凋亡峰（图 5B），同时实验组凋亡细胞数比对照组显著增加（*P* < 0.01），表明乙酸乙酯萃取部位能够诱导A549细胞细胞凋亡。

**2:** 乙酸乙酯萃取部位对A549细胞周期及凋亡率的影响 The effects of ethyl acetate extract on cell cycle and apoptotic rate of A549 cells

Group	Percentage of A549 cells			Apoptotic rate
	G_0_/G_1_	S	G_2_/M	
Control	43.52±3.63	24.33±2.11	32.15±4.72	7.29±2.10
Ethyl acetate	39.33±5.30	32.59±7.68	28.08±2.64	33.86±1.01^**^

**5:**
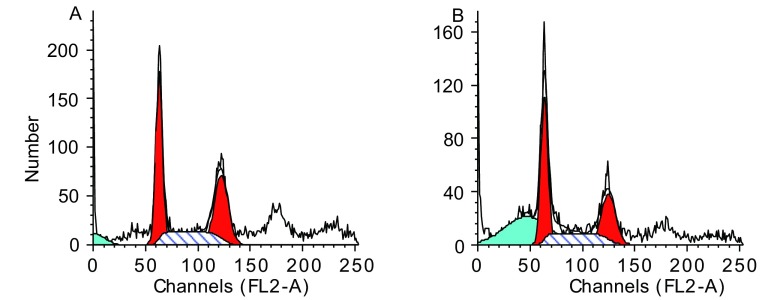
乙酸乙酯萃取部位作用A549细胞流式细胞仪测定结果 Apoptosis of lung cancer cell line A549 induced by ethyl acetate extract. A: Control group; B: Ethyl acetate extract group.

### 2.6 Hoechst 33258染色检测细胞凋亡

经乙酸乙酯萃取部位不同浓度作用72 h后，用Hoechst 33258染色，荧光显微镜结果显示（图 6），对照组细胞所发荧光较弱，较均匀，经乙酸乙酯萃取部位处理后，细胞数量减少，出现有高亮蓝色的典型凋亡小体，其细胞皱缩、细胞核固缩、碎裂、发泡、体积变小，荧光强度增高。说明乙酸乙酯萃取部位有明显诱导A549细胞凋亡的作用。

**6:**
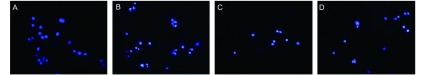
不同浓度乙酸乙酯萃取部位诱导A549细胞凋亡的形态改变（×400） Ethyl acetate extract-induced apoptotic morphologic changes of A549 cells (×400). A: Control group; B: 1 μg/mL ethyl acetate extract group; C: 10 μg/mL ethyl acetate extract group; D: 100 μg/mL ethyl acetate extract group.

### 2.7 Western blot检测结果

与对照组A549细胞相比，不同浓度乙酸乙酯部位处理组的细胞β-actin蛋白表达相当，而细胞的EGFR和ERK1/ERK2蛋白表达量均有下降，其中以100 μg/mL最为明显，10 μg/mL和1 μg/mL次之（图 7），说明乙酸乙酯萃取部位能够下调EGFR-ERK/MAPK信号转导通路。

**7:**
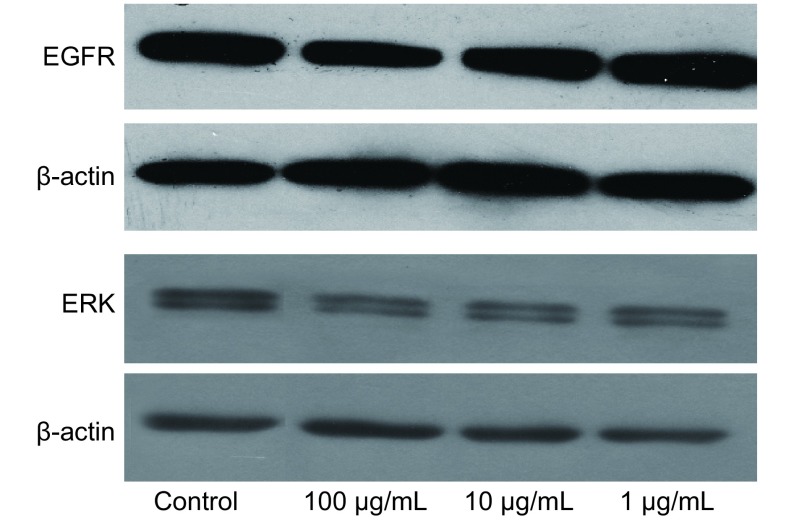
乙酸乙酯萃取部位对A549细胞凋亡相关基因表达的影响 Effects of ethyl acetate extract on expression of apoptosisrelated genes in A549 cells

## 3 讨论

肺癌是世界各国常见的恶性肿瘤之一，在所有恶性肿瘤中增长是最快的，是目前人类癌症死亡的主要原因，并被认为是当今世界对人类健康与生命危害最大的恶性肿瘤。在我国，肺癌占恶性肿瘤发病、死亡的首位。大部分患者就诊时已处于中晚期阶段，现行的常规的治疗手段效果不甚理想。近年来，中医药治疗肺癌在稳定病灶、延长生存时间、改善生存质量等方面发挥了显著的疗效，不仅单用有效，而且可与手术、放疗、化疗联合运用提高临床疗效和减少毒副反应，应用廉价的中药治疗肺癌在临床实践中日益显示出优势。从多靶点综合考虑治疗肺癌是我国中医药治疗肺癌的特色之一，但中药复方的成分复杂、整体层次研究困难，单一成分研究较容易，但又常失去中医药的特色。因此，对于有效部位群的药效相关性研究就显得尤为重要，它在中药现代化研究中起着承前启后的作用。

体外实验是目前抗肿瘤药物研究的重要方法，本研究采用人肺腺癌A549细胞为主要研究对象，对中药复方麦门冬汤合千金苇茎汤有效部位群的体外抑癌作用进行了研究。本研究的检测结果表明，乙酸乙酯萃取部位能有效地抑制A549细胞的增殖，并能诱导肿瘤细胞凋亡。细胞无限制快速生长是恶性肿瘤细胞的主要特征之一，从光镜观察及MTT细胞活力实验证实，乙酸乙酯萃取部位对A549细胞的生长有明显抑制作用，集落形成实验也得出一致的结论，克隆形成率是体外抗癌药物敏感性筛选的重要方法之一，由结果可看出乙酸乙酯萃取部位可以抑制A549细胞克隆形成能力，而克隆形成能力可反应细胞的增殖能力和群体依赖性，克隆形成率的降低说明经乙酸乙酯萃取部位干预后肿瘤细胞群中有增殖能力的干细胞比例减少，从而抑制了肿瘤细胞的增殖。在以上结果的基础上，采用流式细胞术进一步探讨乙酸乙酯萃取部位对A549细胞周期的影响，结果发现实验组细胞在G1峰前出现了明显凋亡峰，提示凋亡细胞数增多，这表明乙酸乙酯萃取部位能够诱导肺腺癌A549细胞发生凋亡。细胞凋亡是基因调控的细胞自主死亡过程，主要表现为染色质凝聚，细胞核固缩，进而核碎裂形成凋亡小体。Hoechst染色细胞核呈致密浓染，或呈碎块状致密浓染。本研究显示，经乙酸乙酯萃取部位干预72 h后的各组细胞，用Hoechst 33258染色荧光显微镜下观察，均能找到细胞核致密浓缩发白发亮的细胞，其细胞核染色质凝集、边移、并发生碎裂、出现凋亡小体，证实乙酸乙酯萃取部位可以诱导A549细胞凋亡，综合以上结果，提示乙酸乙酯萃取部位诱导细胞凋亡是其显著抑制A549细胞增殖的重要途径。

细胞凋亡的机制中，凋亡的细胞因子受体信号转导途径一直被人们所关注，目前认为丝裂原活化蛋白激酶（mitogen activated protein kinase, MAPK）信号传导通路的异常激活与肺癌的发生、发展密切相关^[2]^。细胞外信号调节激酶（extra cellular signal regulated kinase, ERK）是MAPK通路的主要成员，其Raf-MEK-ERK信号转导通路是最具代表性的MAPK信号传导途径之一，也是研究的较为清楚的通路^[3]^，主要介导促有丝分裂和抗凋亡信号，可以通过抑制凋亡促进细胞存活，参与细胞的增殖、分化以及多种代谢功能。研究^[4]^发现，在肺腺癌形成早期阶段ERK通路已被激活。Brognard等^[5]^在对19种组织分型不同的非小细胞肺癌的研究中发现，17种存在ERK通路激活现象，提示在非小细胞肺癌中ERK1/2有促进细胞生存和产生化疗耐药的作用。在转移方面，ERK1/2的激活除了能促进血管内皮生长因子（vascular endothelial growth factor, VEGF）的表达，引起血管生成，促进肺癌的远处转移^[6]^，还参与由糖蛋白聚集素（clusterin, CLU）调节的上皮细胞向间质细胞转变（mesenchymal-to-epithelial transition, MET）和肺腺癌的恶性行为^[7]^。由此可见，ERK蛋白的过表达在肺癌的发生、发展、转移过程中起着重要的促进作用。其主要的作用机制为细胞外生长因子（EGF或TGF-α）与膜受体EGFR结合引起受体二聚化，氨基酸残基磷酸化，从而激活细胞膜内侧的Ras-Raf通路，活化的Ras-Raf可进一步激活MEK-ERK通路，后者进入细胞核作用与c-Jun、c-Fos等多种转录因子，导致细胞增殖或分化。ERK是Ras/Raf/MEK/ERK通路上与细胞核最为靠近的成员，ERK1与ERK2蛋白有90%相同的氨基酸序列，并在体外有相同的作用底物，二者有功能上的重叠。本研究结果显示，乙酸乙酯萃取部位作用于A549细胞后，引起ERK1/ERK2表达下调，说明其激酶活性下降，同时引起上游信号EGFR表达降低，提示EGFR-ERK/MAPK信号通路是乙酸乙酯萃取部位作用靶点之一。

综上所述，本实验证明乙酸乙酯萃取部位在抑制A549细胞增殖的同时使细胞趋于凋亡，其作用机理与ERK信号转导途径密切相关，而乙酸乙酯萃取部位作为细胞外信号分子，作用于细胞后，其受体和信号转导的过程，以及如何启动细胞凋亡基因表达是值得我们进一步研究的课题。因此，对有效部位群及化合物作用肺癌信号转导通路及相关机制的研究值得进一步探讨，同时为丰富中医经典复方有效作用机制提供科学依据。
